# Feeding Low or Pharmacological Concentrations of Zinc Oxide Changes the Hepatic Proteome Profiles in Weaned Piglets

**DOI:** 10.1371/journal.pone.0081202

**Published:** 2013-11-25

**Authors:** Angelika Bondzio, Robert Pieper, Christoph Gabler, Christoph Weise, Petra Schulze, Juergen Zentek, Ralf Einspanier

**Affiliations:** 1 Institute of Veterinary Biochemistry, Freie Universität Berlin, Berlin, Germany; 2 Institute of Animal Nutrition, Department of Veterinary Medicine, Freie Universität Berlin, Berlin, Germany; 3 Institute of Chemistry and Biochemistry, Freie Universität Berlin, Berlin, Germany; University of Navarra School of Medicine and Center for Applied Medical Research (CIMA), Spain

## Abstract

Pharmacological levels of zinc oxide can promote growth and health of weaning piglets, but the underlying molecular mechanisms are yet not fully understood. The aim of this study was to determine changes in the global hepatic protein expression in response to dietary zinc oxide in weaned piglets. Nine half-sib piglets were allocated to three dietary zinc treatment groups (50, 150, 2500 mg/kg dry matter). After 14 d, pigs were euthanized and liver samples taken. The increase in hepatic zinc concentration following dietary supplementation of zinc was accompanied by up-regulation of metallothionein mRNA and protein expression. Global hepatic protein profiles were obtained by two-dimensional difference gel electrophoresis following matrix-assisted laser desorption ionization/time-of-flight mass spectrometry. A total of 15 proteins were differentially (P<0.05) expressed between groups receiving control (150 mg/kg) or pharmacological levels of zinc (2500 mg/kg) with 7 down- (e.g. arginase1, thiosulfate sulfurtransferase, HSP70) and 8 up-regulated (e.g. apolipoprotein AI, transferrin, C1-tetrahydrofolate synthase) proteins. Additionally, three proteins were differentially expressed with low zinc supply (50 mg/kg Zn) in comparison to the control diet. The identified proteins were mainly associated with functions related to cellular stress, transport, metabolism, and signal transduction. The differential regulation was evaluated at the mRNA level and a subset of three proteins of different functional groups was selected for confirmation by western blotting. The results of this proteomic study suggest that zinc affects important liver functions such as blood protein secretion, protein metabolism, detoxification and redox homeostasis, thus supporting the hypothesis of intermediary effects of pharmacological levels of zinc oxide fed to pigs.

## Introduction

Zinc is an essential trace element that plays an important role in many metabolic processes. It acts as a co-factor in metalloenzymes and transcription factors and is involved in DNA replication and RNA transcription, signal transduction, apoptosis or oxidative stress response[1]. In addition, zinc is critical for growth and development as well as for proper immune function and is pivotal for animal and human health (reviewed by Chasapis et al. [2]). Zinc deficiency can result in gastrointestinal, renal and liver diseases; therefore supplementation of zinc has the potential to be a powerful therapeutic agent to prevent such disorders. In young children, for example, dietary supplementation with zinc has been reported to enhance growth and to prevent or treat gastrointestinal disorders [3]. Similar effects could be observed in animals. In pigs, feeding pharmacological (2000–4000 mg/kg) levels of dietary zinc as zinc oxide has been shown to improve performance [4]–[6], and reduce the incidence of diarrhea [7], [8]. The mechanisms are not yet entirely clear, however, possible modes of action have been attributed to the influence of zinc on the gut microbiota [9], [10], epithelial barrier function [11], [12] and/or systemic metabolic effects [13], [14].

Under normal dietary supply, zinc homeostasis is maintained within relatively narrow margins [15]. Zinc is stored in numerous organs with higher levels usually being found in bones, liver, kidney, pancreas, testis, skin, and the retina of the eye [1]. It has been shown that high levels of dietary zinc lead to increased zinc concentration and induction of metallothionein (MT) in various tissues including the liver [16]–[19]. The liver plays a central role in regulation of zinc homeostasis (reviewed by Stamoulis et al. [20]), which in turn is necessary for proper liver function. Due to its key function in the regulation of whole body metabolism of carbohydrates, lipids and proteins, the liver is in the focus of zinc-related health and nutrition research. Gene expression profiling in the liver of piglets revealed the regulation of several key genes when pharmacological zinc levels (2000 mg/kg) were fed [21]. These genes were associated with oxidative stress response and amino acid metabolism. However, whether similar effects can be determined at the protein level is yet unknown.

To our knowledge, this is the first study aiming to determine the influence of pharmacological dietary zinc supply on the global protein expression pattern in the liver of weaned piglets. We used a 2-dimensional differential gel electrophoresis approach (2D-DIGE), which has been previously demonstrated as a powerful tool in nutritional studies [8], [22]. Our hypothesis was that dietary zinc supplementation could modify hepatic protein expression of weaned piglets. Specifically, we identified potential targets in porcine liver that may have the potential to elucidate the cellular and molecular mechanisms of supplemental zinc.

## Materials and Methods

### Animals, feeding and sampling

All procedures involving animal handling and treatment were approved by the local state office of occupational health and technical safety ‘Landesamt für Gesundheit und Soziales, Berlin’ (LaGeSo Reg. No. G0179/09). A total of nine half-sib piglets after weaning at 25+/−1 days of life were used in this study. Experimental setup and samplings were described previously [23]. Briefly, after an adaptation period of 7 days, piglets (n = 3/group) were fed diets containing 50 mg zinc/kg [low zinc (LZn)], 150 mg zinc/kg [normal zinc (NZn)], or 2500 mg zinc/kg [high zinc (HZn)] (Table 1). The dietary zinc levels were adjusted by addition of analytical grade zinc oxide (Sigma, Taufkirchen, Germany) and confirmed by atomic absorption spectrometry (i.e. 50, 156, 2355 mg/kg DM). The animals remained on their respective diets for 14 days before sampling. Following euthanasia, liver samples were immediately snap-frozen in liquid N_2_ and stored at −80°C until further analysis.

**Table 1:** Ingredients and chemical composition of diets used in this study.

Ingredients	g/kg	Chemical composition	g/kg
		Dry matter	885
Optigrain®^1^	500	ME(MJ/kg)	13.5
Barley	100	Crude ash	70
Wheat	70	Crude protein	189
Soybean meal	190	Crude fiber	29
Corn starch/zinc oxide^2^	10.0	Ether extract	39
Whey powder	60	Starch	386
Mineral & Vitamin Premix^3^	10	Lysine	11.8
Soy oil	20	Methionine	4.1
Monocalcium phosphate	16	Threonine	7.1
Limestone	18	Tryptophane	2.3
Salt	2.5	Calcium	11.1
Lysine HC^l^	2.5	Phosphorus	7.7
Methionine	1.0	Sodium	2.7
		Magnesium	1.8
		Zinc mg/kg^4^	50
		Iron mg/kg	119
		Manganese mg/kg	80
		Copper mg/kg	18

1 Optigrain® (DEUKA, Deutsche Tiernahrung, Düsseldorf, Germany), consisting of hydrothermally processed wheat (50%), barley (25%) and corn (25%).

2 Corn starch in the basal diet was partially replaced in the diets containing 150, and 2500 mg/kg zinc with analytical grade zinc oxide (Sigma Aldrich, Deisenhofen, Germany) to adjust for the zinc level.

3 Mineral and Vitamin Premix (Spezialfutter Neuruppin Ltd., Neuruppin, Germany), containing per kg dry matter: 130 g Na (as sodium chloride), 55 g Mg (as magnesium oxide), 700,000 IU Vit A, 120,000 IU Vit D3, 8,000 mg Vit E, 300 mg Vit K3, 250 mg Vit B1, 250 mg Vit B2, 400 mg Vit B6, 2,000 µg Vit B12, 2,500 nicotinic acid, 100 mg folic acid, 25,000 µg biotin, 1,000 mg pantothenic acid, 80,000 mg choline chloride, 5,000 mg Fe (as iron-(II)-carbonate), 1,000 mg Cu (as copper-(II)- sulfate), 6,000 mg Mn (as manganese-(II)-oxide), 45 mg J (as calcium-iodate), 35 mg Se (as sodium-selenite).

4 Analyzed concentration of zinc in the basal diet without ZnO supplementation. The other diets contained 156, and 2355 mg/kg, respectively.

### Liver zinc concentration

Zinc concentration in liver tissue was determined by atomic absorption spectrometry in an AAS vario 6 spectrometer (Analytik Jena, Germany) after hydrolysis of the incinerated tissue in concentrated hydrochloric acid as described by [23].

### Protein extraction and 2-dimensional DIGE analysis

Protein extraction was performed by the addition of 1 ml lysis buffer (9 M urea, CHAPS 2%, biolyte pH 3–10 supplemented with 60 mM DTT, 5 µM PMSF) and protease inhibitor mixture (Calbiochem). Proteins were extracted using a FastPrep FP120 homogenizer (MP Biomedicals) with appropriate lysing matrix tubes followed by incubation for one hour on ice and subsequent centrifugation at 10 000×g, 4°C for 10 minutes. Protein lysates were further purified by a modified TCA-acetone precipitation method (2-D-Clean Up kit, GE Healthcare) and mixed with DIGE labeling buffer (8 M urea, 4% w/v CHAPS, 30 mM Tris, pH 8.5) and the concentration was determined by using a 2-D Quant Kit (GE Healthcare).

A pool consisting of equal amounts of liver tissue from each animal sample was prepared as the internal standard. This internal standard was always labeled with Cy2 and run together with individual liver samples on all gels. The use of this internal standard eliminated errors resulting from artifacts and enables accurate quantitative analysis [24]. Each sample (60 µg total protein) was labeled with 480 pmol appropriate CyDye following the manufacturer's protocol. Two DIGE analyses including 6 gels were performed. Each gel contained the internal standard, one sample obtained from NZn fed piglet and one sample obtained from either HZn or LZn fed animals, respectively. Samples were rehydrated, isoelectrically focused and equilibrated as described previously [22]. SDS-polyacrylamide gel electrophoresis (SDS–PAGE) for the second dimension was carried out in an ETTAN DALT *six* electrophoresis unit (GE Healthcare), first at 0.2 W per gel for 1 h and thereafter at 2 W per gel for further 18 h. Protein spots were visualized by using the Typhoon 9400 laser imager (GE Healthcare) choosing the appropriate wavelength for each CyDye (Cy2 = 520 nm; Cy3 = 580 nm; Cy5 = 670 nm) at a resolution of 100 µm, were cropped and imported into DeCyder V.7.0 software (GE Healthcare). During spot detection by a co-detection algorithm in the software, the estimated number of spots were set at 2500, and the exclude filter was set a slope >1.7 and area <200. The DeCyder differential in the gel analysis (DIA) module was used to process the images from a single gel and enables the pair-wise comparison of each sample to the pooled internal standard. The abundance of each protein spot was determined as a ratio to its corresponding spot present in the internal standard on the same gel. The DeCyder biological variation analysis (BVA) module was used to standardize the ratios across the gels accounting for differences compared with the internal standard.

### Protein identification

Changes of protein expression in response to different Zn feeding detected by 2-DIGE analysis were matched with silver-stained protein patterns (400 µg protein), and the selected spots showing at least a 1.2-fold change in protein expression were excised from the gel. Protein spots were in-gel digested by trypsin (Promega, Germany) as described previously [25]. For protein-identification by matrix-assisted laser desorption/ionization-time-of-flight-mass spectrometry (MALDI-TOF-MS) an Ultraflex-II TOF/TOF instrument (Bruker Daltonics, Bremen, Germany) equipped with a Smart beam™ laser was used. The protein digests were measured in the reflector mode using an α-cyano-4-hydroxycinnamic acid (CHCA) as matrix. For the database search, listed contamination peaks from keratin and autoproteolytic products were excluded for peptide mass fingerprint database search with the Mascot server (www.matrixscience.com) in the NCBInr database and SwissProt. The search was restricted to mammalian sequences and one missed tryptic cleavage was considered. A mass accuracy of 50–100 ppm was used for the searches.

### Immunoblotting

Liver samples were homogenized in DIGE buffer containing protease inhibitor cocktail (Merck Biosciences). Sample proteins (20 µg) and a pre-stained protein-weight marker (Bio-Rad) were resolved by SDS-PAGE in 12% polyacrylamide gels and transferred onto nitrocellulose membranes in Tris-glycine buffer with 20% (v/v) methanol. The membrane was saturated with 5% (w/v) non-fat milk powder (Roth) prepared in Tris-buffered saline containing 0.1% Tween20 (TBST) for 1 h at room temperature and then incubated with primary antibody overnight at 4°C. The primary antibodies employed were mouse monoclonal transferrin antibody (1∶5000, Santa Cruz Biotechnology, Inc.), rabbit polyclonal arginase antibody (1∶400, Santa Cruz Biotechnology, Inc.) and HSP70/72 mouse monoclonal antibody (1∶1000, Enzo Life Sciences). Western blot analysis of MT was performed as described previously [26] using mouse monoclonal MT-antibody (Clone E9, Dako, France) diluted 1∶150. The signals were detected by chemiluminescence with ECL Select (GE Healthcare) according to the manufacturer's instructions.

### Quantitative reverse transcription-polymerase chain reaction (qRT-PCR)

Total RNA was extracted from tissue samples by using an InviTrap Spin Cell RNA Mini Kit (Stratec Molecular, Berlin, Germany) according to the manufacturer's instructions. Duplicates of each total RNA sample (1 µg) was treated with 3.75 µM random hexamers (GE Healthcare, Germany) and 200 U Moloney Murine Leukemia Virus reverse transcriptase (Fermentas, Germany) in a 60 µl reaction mixture in order to generate single-stranded cDNA[27]. Possible genomic DNA contamination was removed prior to reverse transcription by performing a DNase-treatment.

Real-time PCR in the presence of SYBR Green I was performed by using a Rotor-Gene 3000 (Corbett Research, Australia) as described previously [27]. Used primers are indicated in Table 2. The quantity of each specific mRNA was normalized with the program GeNorm [28] and the selected control genes *18S rRNA, GAPDH, SDHA, and RPLA13*. Duplicates were performed of each cDNA sample. The normalized values were used for statistical assessment and for the generation of box and whisker plots. All PCR products were sequenced (GATC, Germany) and showed 100% homology to the known porcine sequences.

**Table 2:** Primer sequences used for real-time PCR analysis.

Gene	Primer sequence	EMBL accession no.	Nucleotide range	Annealing
HSP70	forward 5′-TTGACGCAGGTGTCTTTGAG-3′	X68213	746–974	60°C
	reverse 5′-AAGAGGGAGTCGATTTCCAG-3′			
SERPINH1	forward 5′-CCGTGGGTGTCACTATGATG-3′	NM_001244132	887–1064	60°C
	reverse 5′-AGCTGCTCTTTGGTCAGGAG-3′			
APOA1	forward 5′-CTCCTGGACAACTGGGACAG-3′	X69477	246–532	60°C
	reverse 5′-CTCAGCTTCTCCTGCAGCTC-3′			
ARG1	forward 5′-GGTGGAAGAAGGCCCTACAG-3′	AY 039112	293–635	61°C
	reverse 5′-GGTCGTGGTTGTCAGTGGAG-3′			
EIF4A1	forward 5′-GTTAAGCCGAGGGTTCAAGG-3′	DQ351283	575–813	60°C
	reverse 5′-ACAAGTCACACAGGGTGTCC-3′			
AIFM1	forward 5′-TCCTCCCTGAATACCTCAGC-3′	XM_003135371	1251–1487	60°C
	reverse 5′-GGAAGCCACCGAAATCAGAG-3′			
MT1	forward 5′-GTGAATCCGCGTTGCTCTCTGCT-3′	NM_001001266	7–78	59°C
	reverse 5′-CTGTGGGGCAGGAGCAGTTGG-3′			
GAPDH	forward 5′-ATTCTACCCATGGCAAATTCC-3′	AF017079	484–707	60°C
	reverse 5′- AGGGGCAGAGATGATGACC-3′			
SDHA	forward 5′- CAA ACT CGC TCC TGG ACC TC -3′	DQ845177	869–1131	60°C
	reverse 5′- CCG GAG GAT CTT CTC ACA GC -3′			
RPLA13	forward 5′-CCGTCTCAAGGTGTTCGATG-3′	NM_001244068	328–527	60°C
	reverse 5′-GGATCTTGGCCTTCTCCTTC-3′			
18S rRNA	forward 5′- AAT CGG TAG TAG CGA CGG -3′	AY 265350	254–529	60°C
	reverse 5′- AGA GGG ACA AGT GGC GTT C -3′			

### Statistics

Using Decyder Software proteins were defined as differentially regulated if the observed fold-change calculated as the ratio of the average standardized abundances corresponding to the groups of samples [treatment group (LZn), or (HZn) diet/control group (NZn) diet, respectively] was greater than 1.2 with P-values of less than 0.05 (Student's t-test).

For *qRT-PCR* the normalized mean obtained from the quadruplicates of each RNA sample were analyzed by the Kruskal-Wallis-H-test. In case of significance, the Mann-Whitney-U-test was used for group comparisons. Diagrams and statistical tests of mRNA expression were performed by using SPSS 20.0 (SPSS Inc., USA). The level of significance was set at P<0.05.

## Results

**Figure 1:**
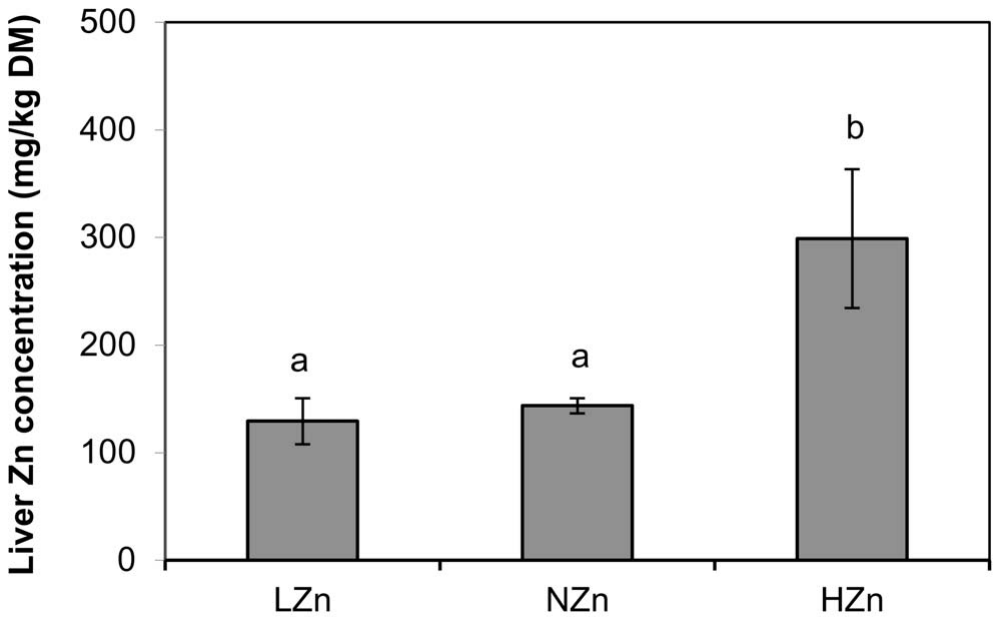
Zinc concentration in the liver of piglets fed LZn (50 mg/kg), NZn (150 mg/kg), or HZn (2500 mg/kg) levels of dietary zinc. ^ab^different superscripts indicate significant (P<0.05) differences.

**Figure 2:**
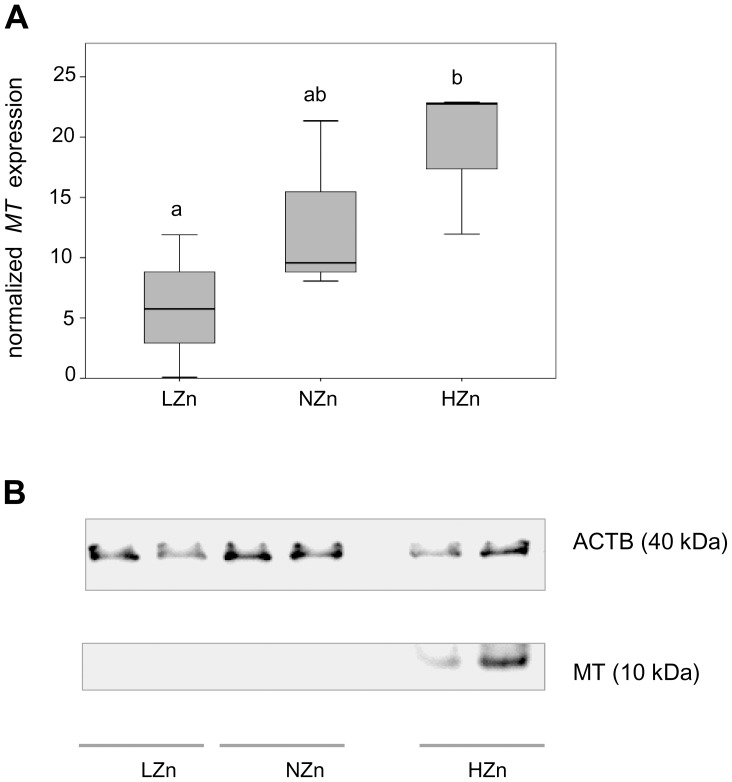
Effects of dietary zinc supplementation on metallothionein expression. Relative metallothionein mRNA levels (**A**) and protein expression (**B**) in the liver of piglets fed LZn (50 mg/kg), NZn (150 mg/kg), or HZn (2500 mg/kg) diet.

**Figure 3:**
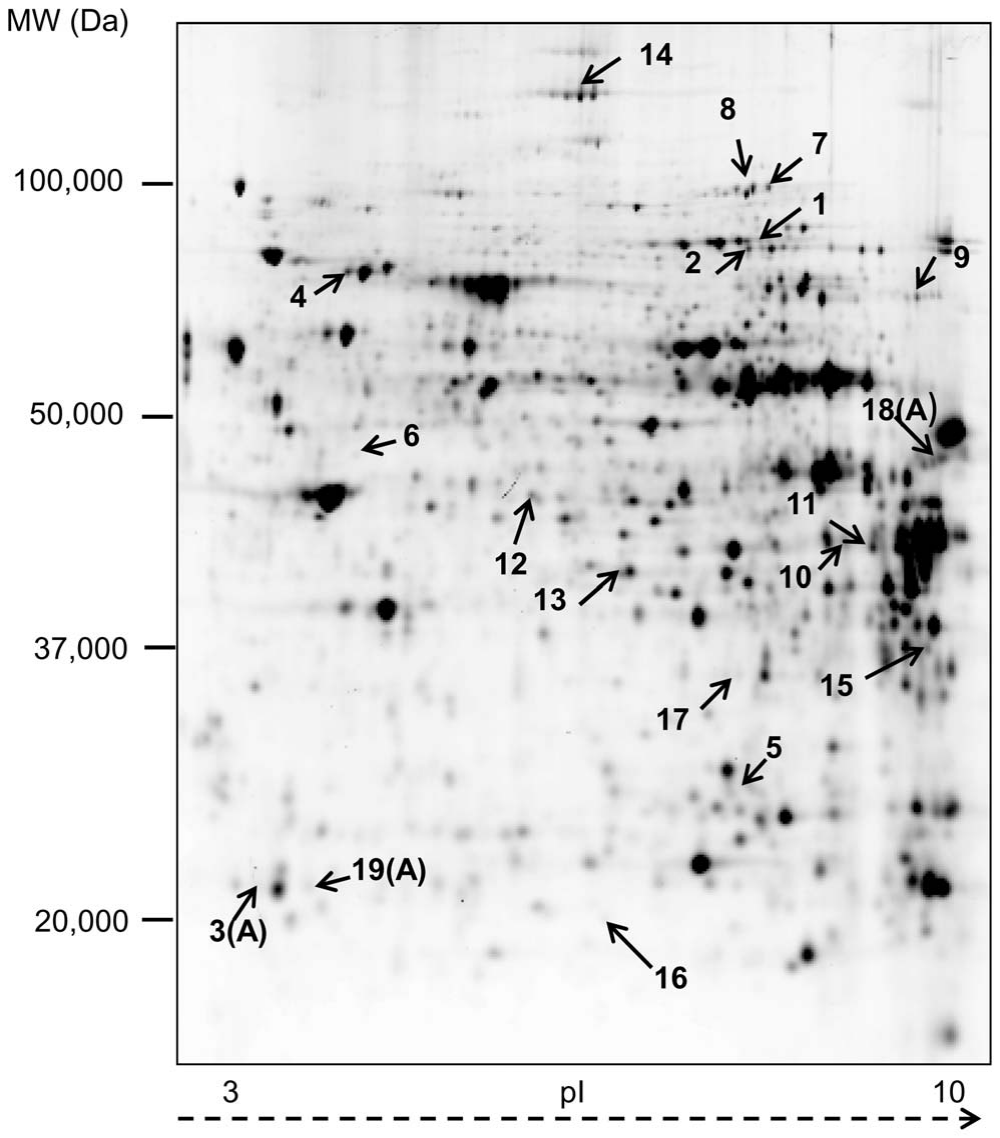
Effects of dietary zinc supplementation on proteomic proteomic profile of porcine liver. Representative 2D gel containing liver proteins from a pool of all analyzed liver samples stained with Cy2. Marked spots represent differentially expressed proteins in hepatic tissue comparing HZn (spot 1–17) or LZn fed piglets (spot 3A, 18A, 19A) with those receiving NZn diet and correspond to the respective spot numbers found in Tables 3 and 4.

All piglets remained in a good health condition throughout the experimental period. As reported previously, performance (feed intake, average daily gain, feed conversion) did not differ between treatments [23]. Liver zinc concentration was higher (P<0.05) with the pharmacological dietary zinc supply (HZn group) but did not differ between NZn and LZn fed piglets (Fig. 1). Similarly, the mRNA level of hepatic MT (Fig. 2A) was significantly higher in HZn fed piglets as compared to the other groups. Induction of MT with pharmacological zinc supply was confirmed by western blot analysis (Fig. 2B). A total of ∼1,500 proteins were detected in hepatic tissue of piglets using the 2D-DIGE approach. Overall, DIGE analysis of the different gels revealed significant changes in expression of 19 proteins, which are assigned to a single representative 2D gel (Fig. 3). Particularly, 17 spots were differentially expressed (>1.2-fold; P<0.05) in the liver of piglets fed HZn diet in comparison with the corresponding spots from the liver of NZn fed piglets (spot 1–17). Among the differentially expressed proteins, 2 proteins were found in more than one spot (Table 3). Among the 15 identified proteins, 7 were up- and 8 down-regulated, respectively (Table 3). Only three proteins were differentially expressed (>1.2fold; P<0.05) between the LZn and NZn group (spot 3A, 18A, 19A). Important biochemical information of all identified proteins corresponding to the numbered spots is summarized in Tables 3 and 4. Transferrin (No. 1; 2) and C-1 tetrahydrofolate synthase (No. 7; 8) were identified in two different spots, respectively. Identified proteins were associated with transport processes (transferrin – TRFE, apolipoprotein A1 – APOA1), signal transduction (eukaryotic initiation factor 4A1 -EIF4A1, apoptosis inducing factor 1-AIFM1), stress response (heat-shock protein 70 – HSP70, endoplasmic reticulum resident protein –ERP 29, HSP47/SerpinH1 precursor-SERPINH1) and metabolic function (aldolase B – ALDOB, glyceral-3-phosphate-dehydrogenase – GAPDH, adenosine kinase – ADK, arginase 1 – ARG1, carbamoyl-phosphate synthase – CPSM, thiosulfate sulfurtransferase- THTR, malate- dehydrogenase – MDH, hypoxanthine-guanine phosphoribosyltransferase – HPRT, Catechol-o-methyltransferase – COMT, C1- tetrahydrofolate synthase – C1TC (Table 3 and 4). HZn supply down-regulated ARG1 and CPSM, while TRFE, APOA1, GAPDH, ALDOB, C1TC and ADK were up-regulated. Notably, compared to the control group (NZn fed piglets) one protein, identified as APOA1, was up-regulated in response to HZn supply (spot 3) and down-regulated in LZn fed piglets (spot 3A).

**Table 3:** List of identified proteins in hepatic tissue of piglets showing differential expression in response to various dietary zinc concentrations.

Name	No.	Accession No.	Av. Ratio	P-value	MW	pI	score
Transferrin	1	gi 136192	+1.81	0.028	78971	6.93	116
	2		+1.55	0.001			136
Apolipoprotein AI	3	gi 164359	+1.45	0.028	30312	5.38	106
Heat shock protein 70	4	gi 178056524	−1.29	0.021	70989	5.24	178
Endoplasmic reticulum resident protein 29	5	ERP29_BOVIN	+1.35	0.035	28845	5.63	56
Eukaryotic initiation factor 4A1	6	IF4AI_BOVIN	−1.21	0.024	46601	5.33	89
C1-tetrahydrofolate synthase,	7	gi 311261216	+1.40	0.026	101771	6.76	280
	8		+1.24	0.024			201
Apoptosis inducing factor 1	9	gi 311276941	−1.22	0.012	66420	9.31	28
Fructose-biphosphate aldolase B-like	10	gi 350579435	+1.20	0.007	27977	8.07	111
Glyceraldehyde-3-phosphate dehydrogenase	11	G3P_PIG	+1.27	0.014	36041	8.51	98
Adenosine kinase	12	ADK_mouse	+1.26	0.014	40466	5.84	60
Arginase 1	13	ARGI1_PIG	−1.27	0.026	35281	6.32	141
Carbamoyl-phosphate synthase, mitochondrial	14	gi 74005321	−1.27	0.005	165634	6.23	172
Malate dehydrogenase	15	MDHM_PIG	−1.26	0.040	36029	8.93	118
Hypoxanthine- guanine phosphoribosyl-transferase	16	HPRT_PIG	−1.54	0.047	24768	6.3	47
Thiosulfate sulphurtransferase	17	gi 311255145	−1.58	0.023	37861	8.58	132
Apolipoprotein A1	3A	gi 164359	−1.90	0.001	30307	5.48	70
Serpin H1-precursor	18A	gi 346421378	+1.55	0.006	46648	8.91	115
Catechol-o-methyltransferase	19A	gi 305855180	+1.28	0.047	30413	5.38	75

**Table 4:** Function of differentially expressed proteins.

Protein name	No.	Predicted protein function
** Transport function**		
Transferrin	1/2	Transport of iron, antioxidant
Apolipoprotein A1	3/3A	Transport of cholesterol, HDL-metabolism
** Signal transduction**		
Eukaryotic initiation factor IF4A1	6	Initiation of translation
Apoptosis inducing factor	9	Initiation of apoptosis
**Stress response function**		
Heat shock protein 70	4	Chaperone, protein folding
Endoplasmic reticulum resident protein 29	5	Chaperone, processing and transport of proteins
Serpin H1 precursor	18A	Chaperone, collagen binding protein
** Metabolic function**		
Fructose-biphosphate aldolase	10	Glycolysis
Glyceraldehyde-3-phosphate dehydrogenase	11	Glycolysis
C-1 tetrahydrofolate synthase	7/8	C1-metabolism
Adenosine kinase	12	Phosphate transfer, methylation
Arginase 1	13	Urea cycle
Carbamoylphosphate synthase	14	Urea cycle
Malate dehydrogenase	15	Tricarboxylic acid pathway
Hypoxanthine-guanine phosphoribosyl-transferase	16	Purine salvage pathway
Catechol-o-methyltransferase	19A	Methylation of DNA
Thiosulfate sulfurtransferase	17	Sulfur metabolism, detoxification

**Figure 4:**
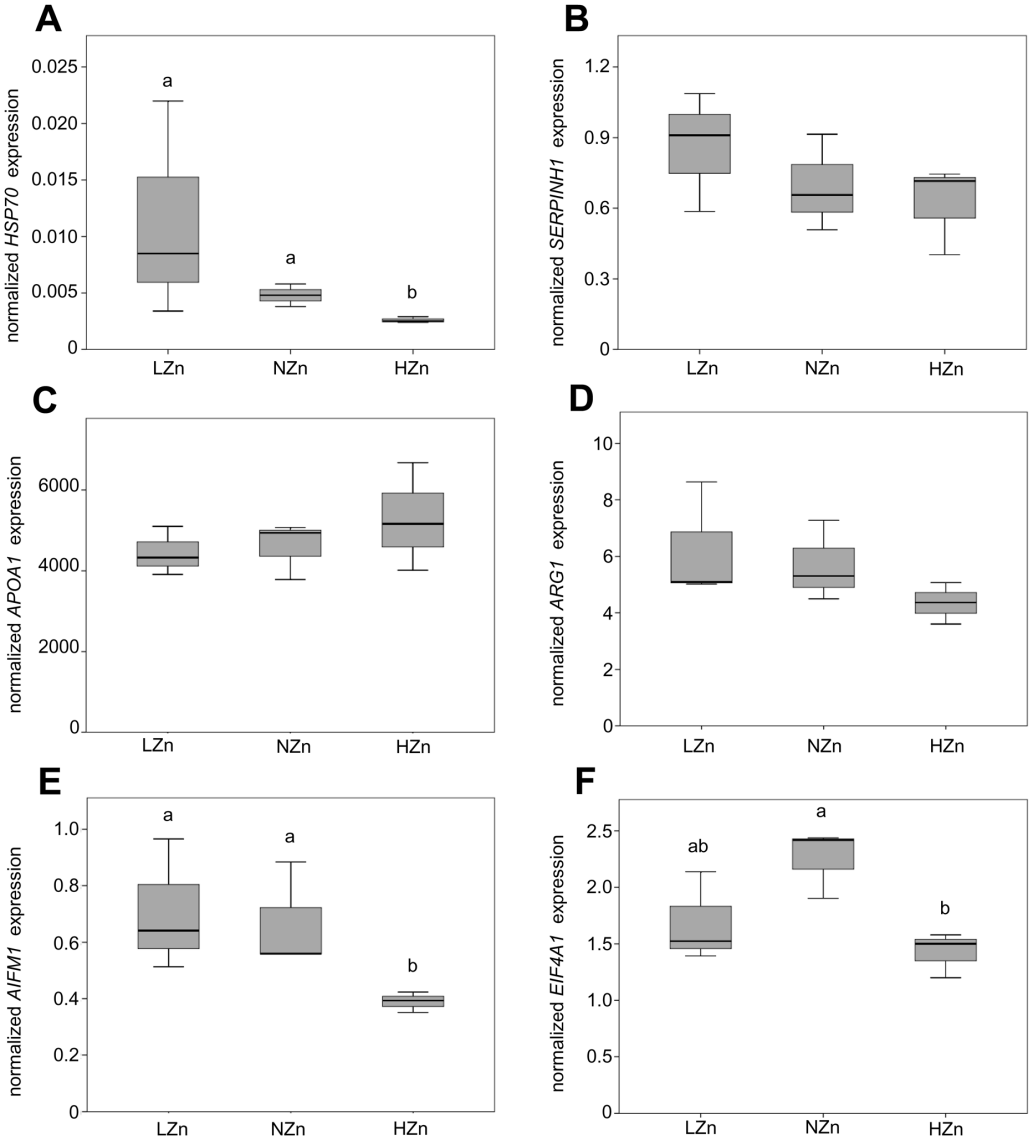
Effects of dietary zinc supplementation on relative mRNA level of differentially expressed liver proteins. Relative mRNA level of *HSP70* (**A**), *SERPINH1* (**B**), *APOA1* (**C**), *ARG1* (**D**), *AIFM1* (**E**), and *EIF4A1* (**F**) in hepatic tissue of piglets fed LZn (50 mg/kg), NZn (150 mg/kg), or HZn (2500 mg/kg) levels of dietary zinc. Different superscripts indicate significant (P<0.05) differences.

Differential expression of selected proteins (i.e. SERPINH1, HSP70, APOA1, ARG1, AIFM1, EIF4A1) was confirmed at the mRNA level (Fig. 4A–F). The mRNA level for *HSP70*, *EIF4A1* and *AIFM1* was decreased (P<0.05) with HZn supply compared with NZn fed animals. *ARG1* showed the same tendency (P = 0.07). *SERPINH1* was up-regulated and *APOA1* was down-regulated in the LZn-feeding group, but failed to reach significance.

**Figure 5:**
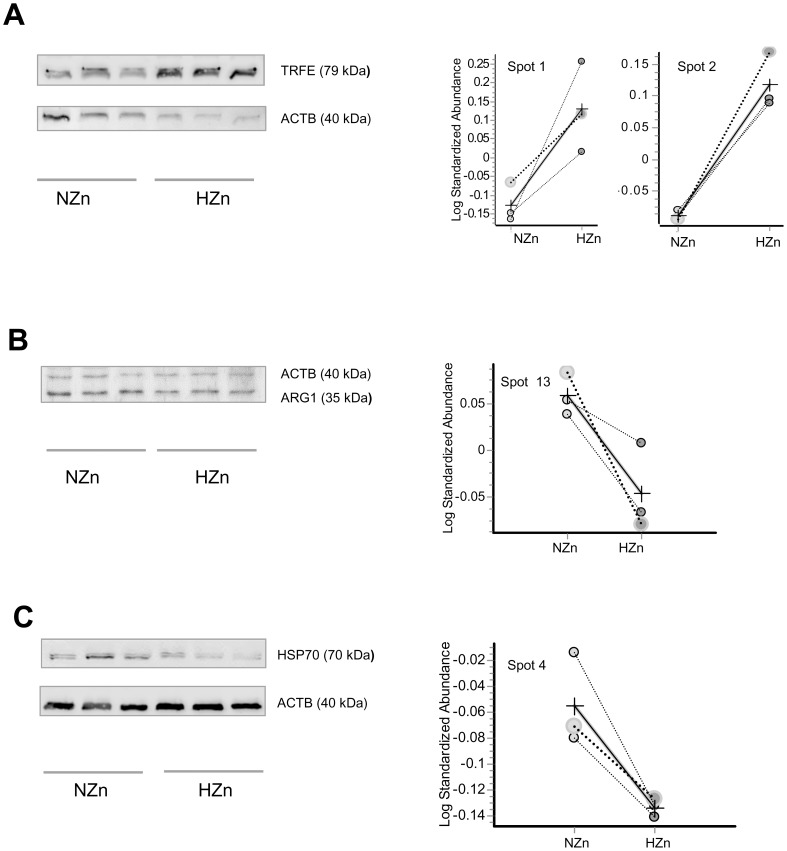
Validation of differential protein expression by Western blotting. Western blot demonstration of differentially expressed proteins TRFE (**A**), ARG1(**B**), and HSP70(**C**) in the liver of piglets fed NZn (150 mg/kg), or HZn (2500 mg/kg) diets.

Western blotting was used to verify differential protein expression in hepatic tissue between piglets fed NZn and HZn diet (Fig. 5A–C). One representative protein out of three different functional groups (see Table 4), namely TRFE, ARG1 and Hsp70 was examined. On the right side of Fig.5 the protein abundances of these selected proteins (detected in each gel and calculated by the BVA module of the Decyder software) are indicated. Increased protein abundance in the liver of HZn-fed piglets was confirmed for TRFE (Fig. 5A), whereas reduced protein levels were confirmed for ARG1 (Fig. 5B) and HSP70 (Fig. 5C).

## Discussion

The current study reveals that feeding pharmacological dietary zinc not only affects the hepatic zinc concentration in weaned piglets but, moreover, changes the global protein expression pattern. Zinc is one of the essential dietary elements for normal growth and development and innate immune function [1]. Feeding pigs with pharmacological levels of zinc to the diet of weanling piglets can enhance growth performance [17], [29], [30]. Proposed mechanisms for the enhanced growth of piglets involve MT as one candidate protein [17]–[19]. This was further underlined by our study demonstrating induced hepatic MT expression in response to HZn dietary zinc supply at transcript as well as protein level. MTs are a family of low molecular weight proteins (0.5–14 kDa) with high cysteine content that could regulate the availability of cellular zinc [31]. Thus, MT-level may be involved in triggering zinc signaling pathways, particularly in balancing redox homeostasis and metabolism. It has been demonstrated that the induction of MTs by zinc in the liver of mice or rats could play an important role in protection against oxidative stress [32], [33]. Although we did not observe significant differences in growth rate and feed intake in our approach, metabolic changes in the liver with HZn diets can be assumed. Interestingly, gene expression profiling in porcine hepatic tissue identified genes involved in reducing oxidative stress and amino acid metabolism [21]. Using a proteomic approach, we were able to even identify further targets of zinc in the liver providing a possible basis to unravel molecular mechanisms involved in the response HZn feeding. To our knowledge, the current study provides first evidence of a differential hepatic proteomic profile in pigs in response to pharmacological level of dietary zinc. The identified proteins are multifunctional and involved in important cellular processes.

### Transport proteins

Two transport proteins were regulated in response to dietary zinc, namely APOA1 and TRFE. Particularly, the abundance of APOAI was lower in pigs fed LZn diet when compared to NZn-fed animals (P<0.05) and vice versa this protein was up-regulated in liver of HZn fed group. APOAI is mainly synthesized in the intestine and liver and is the major protein component of plasma high-density lipoprotein (HDL) particles. In growing rats, it has been demonstrated that Zn deficiency results in decreased serum HDL and plasma glucose, whereas cholesterol was increased [34], [35]. Accordingly, a down-regulation of hepatic APOAI mRNA abundance has been reported in rats and hamsters [36] and in Hep G2 cells [37], [38] under zinc-deficient conditions. On the other hand, Zn supplementation resulted in an increased cellular APOA1 mRNA abundance [37] and a concomitant increase of MT [39]. In accordance with these published results our data suggest that the Zn status regulates hepatic APOA1 expression in pigs.

High zinc level increased the expression of TRFE. TRFEs are iron-binding transport proteins that are involved in the transport of iron from absorption sites to those of storage and utilization (reviewed by Theil [40]). TRFE is not only an iron binding protein, but also binds zinc [41] and has functions in both, iron and zinc absorption and transport [42]. However, no difference in iron concentrations of the liver was detected in this study (data not shown). Thus, the up-regulation of TRFE might be a response to high hepatic zinc concentration. Other zinc binding proteins such as MT were also highly up-regulated in the liver. Additionally, due to its ability to chelate free iron, transferrin may have an important role as antioxidant [43].

### Apoptosis

Our finding that feeding the HZn diet down-regulates the expression of AIFM1 in porcine liver is in line with other studies reporting zinc as a potent inhibitor of apoptosis [44]. Particularly, zinc supplementation has been shown to inhibit hepatic apoptosis in mice [45] and on the other side zinc deprivation induces apoptosis in several cell lines including HepG2 cells [46], [47].

### Stress proteins

Increased expression of HSPs is considered as universal response to stress, which plays an important role in protecting cells from harmful environmental conditions as reviewed in Ref. [48], including pigs [49]. However, it is also known that these heat-shock proteins possess a more global role in cell metabolism, making it complicated to address their specific action. Heat shock proteins like HSP70 act as cellular chaperones and are involved in different cellular functions, such as, protein folding and assembly or reassembly. Particularly, HSP70 is known to inhibit the aggregation of nascent or miss-folded proteins to regulate protein degradation and to help in translocation of proteins between different cellular compartments [50], [51]. Previously our group has reported an up-regulation of HSP70 expression in porcine intestinal cells exposed to high concentrations of zinc [52]. This is consistent with data on mice, indicating enhanced HSP70 expression in the gastrointestinal tract in response to high dietary zinc [53]. Interestingly, here we found a down-regulation of liver HSP70, which could be confirmed by immunoblotting and was further underlined by decreased *HSP70* mRNA. This is in accordance with the results of a gene expression study in hepatic tissue of newly weaned pigs fed with 2000 mg Zn/kg [21]. These authors speculated that zinc induced chaperone expression will be counteracted at higher zinc concentration by inhibiting their expression, previously discussed by other authors [54]. Recently published results indicate different heat shock response in different organs and tissues of pigs after transportation. [55]. Therefore it is possible that heat shock expression in different cell types and organs could be regulated through distinct mechanisms in a complex manner at both transcriptional and translational levels. However, the precise function of down-regulation of HSP70 in porcine liver in response to zinc needs further investigations. Interestingly, the ERP29, another putative chaperone, was found up-regulated in response to high zinc concentrations. ERP29 is known to self-associate into 51 kDa dimers (also seen in Fig. 3) and to lack redox enzyme properties, such as disulphide-editing, that are typical for other chaperones [56]. The functional role in the liver may be related to its important role in the early secretory pathway facilitating processing and transport of proteins [57]. The up-regulation of ERP29 under high dietary zinc possibly occurs through involvement of zinc regulatory transcription factors including Sp1 [58] that have been reported to be member of highly related zinc finger proteins [59]. It should be mentioned, that zinc deficiency is also associated with stress [35]. This suggestion can be underlined by the results of our study. As compared with NZn group, the LZn feeding of 50 mg Zn/kg affects the expression of the SERPINH1 (HSP47) precursor. The serpins are a family of serin proteinase inhibitors with pleiotropic functions extending from protease inhibition to hormone transport and regulation of chromatin organization [60]. Interestingly, SERPINH1 (Hsp47) is a stress inducible collagen binding protein acting as protein-specific “manager” assisting the synthesis, post-translational modification and transport of (pro)-collagen without protease inhibitory activity [60]. Its induction was reported by liver damage and markedly up-regulated during progression of liver fibrosis [61], [62].

### Metabolism

The liver plays the major role in the metabolism of nutrients and other dietary substances [63]. Accordingly, 10 differentially expressed proteins involved in various metabolic pathways were identified (Tab. 3 and 4). Particularly, two enzymes of the glycolysis were found to be up-regulated by high dietary zinc concentrations, namely ALDOB and GAPDH. These enzymes have been recently characterized as zinc-binding proteins in human hepatocytes [64]. Some evidences indicate that high zinc concentrations stimulate glycolysis as shown on isolated rat hepatocytes and primary mouse hepatocytes [65], [66]. Furthermore this effect was markedly diminished in hepatocytes from MT-null (-/-) mice suggesting a close link between zinc induced increase of hepatic MT and increased glycolysis. Interestingly, our study reveals a down-regulation of the MDH by zinc. However, this inhibitory effect of zinc could be also demonstrated in chick-embryo hepatocytes [67] and may be underlined by studies on the isolated malic liver enzyme [68]. Furthermore, the expression of two enzymes of the urea-cycle was moderately decreased. This corresponds with an exponential decline in luminal ammonia concentration [23] and might suggest an adaptive depression of the urea cycle in the liver. The one-carbon metabolism is connected with other pathways like biosynthesis of nucleic acids, conversion of creatine and amino acids as well as in vitamin metabolism. Furthermore, zinc is also essential for synthesis of coenzymes that mediate biogenic-amine synthesis and metabolism [69]. Accordingly, in our study the expression of the C1TC was up-regulated by zinc. Notably, it has been reported that folate deficiency enhances perturbations in methionine metabolism and DNA damage while promoting alcoholic liver injury [70]. This in turn could be suppressed by zinc supply [71]. Another enzyme, the ADK playing an important role in the maintenance and balance of methylation reactions, was found to be up-regulated by high dietary zinc in the liver. The HPRT converts guanine to guanosine monophosphate, and hypoxanthine to inosine monophosphate. This enzyme plays a central role in the generation of purine nucleotides through the purine salvage pathway. In rats, which received a zinc-deficient diet, an enhanced activity of the HPRT was observed [72]. Accordingly, we found a down-regulation of this enzyme in response to high dietary zinc. The porcine THTR is found with increased activities in liver and kidney [73] and suggested to be involved in cyanide detoxification [73], [74]. However, this enzyme may be involved in other functions, including energy and sulfur metabolism; a function as thioredoxin oxidase and involvement in proliferation and progression of cell cycle are further discussed (reviewed by Cippolone et al. [75]). Rhodanese was found to be inhibited by zinc by more than 70%, implying an interaction of zinc with the sulfhydryl groups of the enzyme catalytic sites [76], [77]. Our study provides evidence that this enzyme is related to zinc status in liver but clearly requires further investigations to define its exact biological function.

In summary, high dietary zinc supplementation induced an increase in the levels of both hepatic zinc and MT expression, and altered the protein expression profiles. Using a 2D-DIGE proteomic approach, we were able to describe for the first time significant protein changes in the liver, despite of a limited piglet number. We provide initial insights on the complex regulatory network of zinc-induced protein expression pattern alterations in the liver. The identified proteins were involved in transport, stress response, metabolism, apoptosis and cellular signaling. Further work is needed to determine their functional relevance and to clarify whether changes in the level of the above mentioned proteins are due to a direct effect of zinc supplementation or a consequence of its interaction with other target(s).
